# Implication of Adipogenesis-Coupled CRMP2 Functional Profile in Metabolic Homeostasis and Imbalance

**DOI:** 10.3390/biomedicines10102603

**Published:** 2022-10-17

**Authors:** Yih-Hsin Chang, Shu-Wen Chang, Wei-Ting Hsu, Ching-Ping Yang, Yu-Li Lo, Chun-Jung Chen, Hui-Fang Tsai, Ming-Yuh Shiau

**Affiliations:** 1 Department of Biotechnology and Laboratory Science in Medicine, National Yang Ming Chiao Tung University, Taipei 112, Taiwan; 2 Department of Nursing, College of Nursing, Hungkuang University, Taichung 433, Taiwan; 3 Department of Medical Technology, Jenteh Junior College of Medicine, Nursing and Management, Miaoli 356, Taiwan; 4 Department and Institute of Pharmacology, National Yang Ming Chiao Tung University, Taipei 112, Taiwan; 5 Department of Medical Research, Taichung Veterans General Hospital, Taichung 407, Taiwan; 6 Department of Medical Laboratory Science and Biotechnology, China Medical University, Taichung 404, Taiwan; 7 Department of Medical Laboratory and Biotechnology, Chung Shan Medical University, Taichung 402, Taiwan; 8 Clinical Laboratory, Chung Shan Medical University Hospital, Taichung 402, Taiwan

**Keywords:** adipogenesis, collapsin response mediator protein 2, lipid metabolism, glucose

## Abstract

Our previous studies demonstrated that collapsin response mediator protein 2 (CRMP2) is associated with obesity and, in addition, that hyperglycemia-suppressed CRMP2 augments malignant traits of colorectal cancer and is associated with advanced tumor stage. Regulation of CRMP2 profile was further explored in this study using 3T3-L1 pre-adipocyte adipogenesis as a study model for illustrating the roles of CRMP2 in metabolic homeostasis. Hyperglycemia inhibited expression of CRMP2, adipogenic machinery and adipocyte markers. CRMP2 displayed f-CRMP2 (62~66 kDa) and s-CMRP2 (58 kDa) isoforms at the growth arrest phase. Expression of s-CRMP2 was coupled with the mitotic clonal expansion (MCE) phase to direct cell proliferation and rapidly down-regulated in post-mitotic cells. In the late differentiation phase, f-CRMP2 was co-localized with tubulin in the cortical area. Insulin-enhanced CRMP2-glucose transporter 4 (GLUT4) co-localization and CRMP2 puncta on lipid droplets (LDs) suggested participation of CRMP2 in GLUT4 translocation and LD fusion. Collectively, the CRMP2 functional profile must be finely controlled to adjust cytoskeletal stability for meeting dynamic cellular needs. Manipulating the s-CRMP2/f-CRMP2 ratio and thus the cytoskeleton dynamics is anticipated to improve glucose uptake and insulin sensitivity. In summary, our data provide molecular evidence explaining the functions of CRMP2 in physiological, pathological and disease progression in metabolic homeostasis and disorders related to metabolic abnormalities, including cancer.

## 1. Introduction

Obesity, caused by long-term overnutrition, is highly associated with the onset of type 2 diabetes mellitus (T2DM) with hyperglycemia and insulin resistance as the major characteristics. Insulin resistance in obesity is the outcome manifested by a complex interplay composed of increased fatty acid flux, altered secretory pattern of adipocyte-derived cytokines (adipokines) by the hypertrophic adipose tissue, chronic tissue inflammation, etc. Thus, delicately tuned interactions among various systems are required for maintaining energy balance and metabolic homeostasis.

Insulin is the predominant endocrine hormone that regulates energy balance by promoting anabolic metabolism. For adipose tissue, insulin triggers translocation of intracellular glucose transporter 4 (GLUT4) to plasma membrane for facilitating glucose uptake [1]. The net effects of insulin on adipocytes are to promote lipid accumulation and inhibit lipolysis for increasing lipid deposits via promoting lipogenesis [2].

GLUT4 is mainly expressed in adipose tissue and muscle [3]. The majority of GLUT4 molecules are packaged into cytosol compartments designated as GLUT4-storage vesicles (GSVs) in pre-prandial state. Post-prandial insulin triggers GSV exocytosis for translocating intracellular GLUT4 to plasma membrane and facilitating glucose uptake [4]. Disrupting the actin network inhibits GLUT4 translocation and inefficient glucose uptake, which eventually leads to insulin resistance, indicating that cytoskeleton remodeling is critical for GSV translocation and fusion with plasma membrane [5,6]. Our previous study revealed that collapsin response mediator protein 2 (CRMP2), one of the major microtubule dynamics-mediating molecules, displays multiple regulatory activities in adipocyte differentiation (adipogenesis) and lipid deposits [7] via modulating cell proliferation in the initial mitotic clonal expansion (MCE) phase, critical adipogenic transcription factors and lipid-synthesizing enzymes. Notably, GLUT4 translocation and glucose uptake are disturbed in CRMP2-silencing cells by accelerated actin de-polymerization in an insulin-independent manner. Moreover, CRMP2 is significantly increased in adipose tissue of high fat diet-induced obesity (DIO) mice, indicating CRMP2 is associated with obesity.

Our most recent report disclosed that hyperglycemia suppresses CRMP2 expression/activity and augments colorectal cancer (CRC) malignant traits; thus, CRC patients with diabetic comorbidity (CRC-DM) have significantly lower CRMP2 levels than CRC subjects and manifest advanced tumor stage [8]. We conclude that hyperglycemia leads to enhanced cell proliferation and cytoskeleton flexibility via promoting actin de-polymerization, and thus endows CRC cells with higher metastatic potential by downregulating CRMP2 profile and contributes to CRC disease progression. Our findings provide molecular evidence elucidating that diabetic hyperglycemia exacerbates disease progression and poor prognosis in cancers via mediating cytoskeleton stability [9,10,11].

Taking the above evidence together, CRMP2 levels and activity are crucial and must be finely tuned for maintaining cytoskeleton dynamics and flexibility of mechanical strength in response to cellular metabolic needs. In this context, the regulatory mechanism of CRMP2 expression and effects of glucose on CRMP2 were explored using 3T3-L1 pre-adipocyte adipogenesis as a study model for further illustrating the roles of CRMP2 in metabolic homeostasis.

## 2. Materials and Methods

### 2.1. Reagents

Antibodies against CRMP2, Thr514 phosphorylated CRMP2 (pCRMP2), CCAAT-enhancer-binding protein-alpha (C/EBPα), peroxisome proliferator-activated receptor gamma (PPARγ), and fatty acid binding protein-4 (FABP4, or aP2) were purchased from Cell Signaling Technology (Danvers, MA, USA); glucose transporter 4 (GLUT4) from Novus Biologicals (Centennial, CO, USA); and GAPDH, anti-tubulin and EHD1 from GeneTex, Inc. (Irvine, CA, USA). ECL reagent was purchased from Calbiochem (Merck Millipore, Billerica, MA, USA); Trizol Reagent from Life Technology (Carlsbad, CA, USA); 3-isobutyl-methylxanthine (IBMX), dexamethasone (Dex), insulin and β-actin from Sigma (St. Louis, MO, USA); BODIPY^®^ 493/503 from Thermo Fisher Scientific (USA); and protein A Sepharose CL-4B from Invitrogen (Carlsbad, CA, USA).

### 2.2. Cell Culture of 3T3-L1 Adipocytes and Oil Red O Staining

3T3-L1 fibroblasts were maintained in DMEM containing 10% calf serum (Hyclone Laboratories, South Logan, UT, USA) in an atmosphere of 5% CO_2_ at 37 °C as previously described [7,8,12,13,14,15]. Two days after fibroblasts had reached confluence, differentiation was induced by adding DMEM containing 0.5 mM IBMX, 10 μg/mL insulin and 1 μM Dex (MDI cocktail), and 10% fetal bovine serum (FBS) for 48 h. Cells were cultured in DMEM supplemented with 10% FBS and 5 μg/mL insulin for the next 6 days. Differentiation efficiency, cell morphology features and intracellular lipids were assayed by Oil Red O (ORO) staining on day 8. Briefly, cells were stained with ORO (0.6% dissolved in isopropanol and water, 6:4) for 30 min, then washed with distilled water. For quantification, lipid amounts were determined by measuring the absorbance of isopropanol-eluted dye at 490 nm.

### 2.3. RNA Analysis and mRNA Expression

Levels of mRNA were investigated by RT-PCR. At the indicated time, total RNA was harvested using Trizol reagent and converted to cDNA with 200 U Moloney murine leukemia virus reverse transcriptase in 30 μL of buffer containing 0.5 mM dNTPs and 0.1 μg oligo(dT)18 primer. Then, cDNA was amplified with Thermus aquaticus DNA polymerase using specific primers (CRMP2: 5′-ATTCCAGCTGACGGATTCCCAGAT-3′ and 5′-TGATGTCACCATTCTCTGCGTGGA-3′; GAPDH: 5′-ACCACAGTCCATGCCATCAC-3′ and 5′-TCCACCACCCTGTTGCTGTA-3′). Following amplification, PCR products were separated on 2% agarose gel, stained with ethidium bromide and visualized under UV light.

### 2.4. Protein Analysis

Protein expression profiles were investigated by Western blot. At the indicated time, cell lysates were obtained by the addition of 100 mL SDS sample buffer and then incubated at 100 °C for 4 min, fractionated by 10% SDS-PAGE and electrophoretically transferred to a nitrocellulose membrane (Hybond ECL, Amersham Pharmacia Biotech). The membranes were first incubated in TBS-T (25 mM Tris-HCl at pH8.0, 125 mM NaCl and 0.01% Tween-20) containing 5% non-fat dry milk (Difco Laboratories, Detroit, MI, USA), then respectively incubated with specific primary antibody. The blots were incubated with the secondary antibody for 1 h and developed by the enhanced chemiluminescence system.

### 2.5. Subcellular Fractionation Studies

Lipid droplets (LDs) and cytosolic and crude membrane fractions were isolated by centrifugation of protein extracts from mature adipocytes by sucrose gradient centrifugation as described in [16] with modifications. Cells were rinsed with PBS and resuspended in lysis buffer (pH 7.4) containing 20 mM Tris-HCl, 1 mM EDTA, 10 mM NaF, 1 μg/mL leupeptin and 1 μg/mL aprotinin. Cell lysates were harvested after being disrupted in tissue homogenizer on ice, then centrifuged at 1000× *g* for 10 min at 4 °C. Supernatants were transferred to a 15 mL ultracentrifuge tube, mixed with an equal volume of lysis buffer containing 60% sucrose, and overlaid with 5 mL of 5% sucrose buffer and 5 mL sucrose-free lysis buffer. After centrifugation at 28,000× *g* at 4 °C for 30 min, protein distribution in fractions of 600 μL aliquot was analyzed by Western blotting.

### 2.6. Co-Immunoprecipitation Assay

Cell lysates were incubated with 1 μg anti-EDH1 antibody overnight at 4 °C and captured by protein A/G agarose for 2 h. After washing with RIPA buffer (50 mM Tris-HCl pH7.4, 150 mM NaCl, 1 mM EDTA, 1 mM EGTA, 1 mM Na_3_VO_4_, 1% NP-40, 0.25% sodium deoxycholate, 0.1% SDS, protease- and phosphatase- inhibitor cocktail), immunoprecipitates were eluted in sample buffer, denatured, and subjected to Western blotting with the indicated antibodies.

### 2.7. Immunostaining Analysis and Microscopic Observation

Cells were fixed with 3.7% formaldehyde for 15 min, treatment with 0.5% Triton X-100 for 15 min at room temperature, 5% BSA for 30 min at room temperature followed by incubation with specific primary antibody overnight at 4 °C. After washing, the samples were incubated with goat anti-rabbit IgG-conjugated DyLight TM 594 (Jackson ImmunoResearch Laboratories, West Grove, PA, USA) together with BODIPY (Molecular Probes, Eugene, OR, USA) or appropriate secondary antibody for 1 h at room temperature. Cells were then mounted with Gel/Mount containing DAPI (Molecular Probes). Images were taken by using the Zeiss LSM 700 confocal fluorescence microscope system with a 63× objective lens.

### 2.8. Statistical Analysis

Each experiment was carried out at least three times. All values are presented as mean ± SEM. For statistical analysis, the *p* value was calculated using a two-tailed unpaired Student’s *t*-test with *p* < 0.05 considered as statistically significant.

## 3. Results

### 3.1. CRMP2 Expression Profile in Undifferentiated Pre-Adipocytes and Adipogenesis

**Figure 1:**
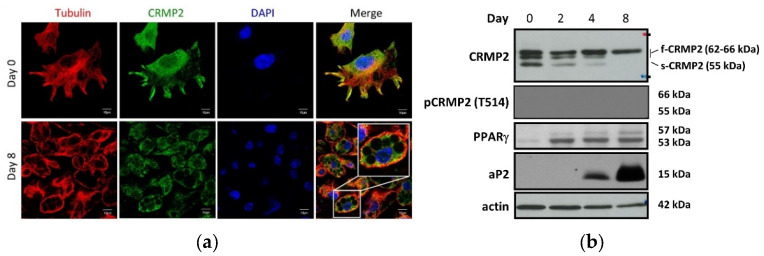
CRMP2 expression pattern in undifferentiated pre-adipocytes and during the process of adipogenesis. 3T3-L1 pre-adipocytes were induced to enter differentiation scheme by MDI cocktail on day 0. (**a**) Distribution of CRMP2 and α-tubulin before (day 0, upper panel) and after (day 8, lower panel) differentiation was visualized by confocal imaging (scale bar = 10 μm). (**b**) Expression of CRMP2 and adipocyte markers was temporally examined by Western blotting at the indicated time (*n* = 6).

Intracellular distribution of CRMP2 was first analyzed in undifferentiated pre-adipocytes and terminally differentiated mature adipocytes by immunostaining. In pre-adipocytes, cells exhibited typical fibroblast morphology with multiple protrusions (Figure 1a, upper panel). CRMP2 was mainly localized in the peri-nuclear region and co-localized with α-tubulin at the leading edge of protrusions. After being allowed to enter differentiation for 8 days, the cells transformed to round-shape adipocytes containing multiple lipid droplets (LDs) residing in cytoplasm (Figure 1a, lower panel). CRMP2 was evenly dispersed in cytosol except for the areas occupied by LDs, and co-localized with α-tubulin in the cortical area. Paralleled with our previous report [7], multiple CRMP2 subtypes including full-length CRMP2 (f-CMRP2, 62~66 kDa) and smaller CRMP2 fragments (s-CMRP2, 58 kDa) were identified in the pre-adipocytes on day 0 (Figure 1b). Once the cells entered differentiation, s-CRMP2 was rapidly decreased along with differentiation progression and barely detected in the terminal differentiated mature adipocytes on day 8.

### 3.2. Transcriptional and Post-Transcriptional Regulation of CRMP2 during Adipogenesis

**Figure 2:**
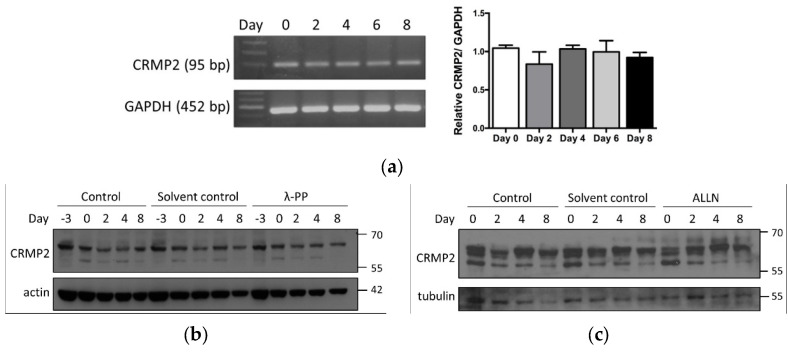
Transcriptional control and post−transcriptional modification of CRMP2 during adipogenesis. (**a**) CRMP2 mRNA levels were examined by RT-PCR (*n* = 3). (**b**,**c**) 3T3-L1 pre-adipocytes were subjected to differentiation in the presence of (**b**) λ-PP phosphatase or (**c**) calpain inhibitor ALLN treatment. Cell lysates were collected at the indicated time and subjected to protein analysis by Western blotting (*n* = 4).

It was intriguing to probe if transcriptional and post-translational regulation contributed to the identified CRMP2 expression pattern. CRMP2 mRNA levels remained rather consistent during the entire period (Figure 2a), suggesting transcriptional regulation was not the underlying factor. For post-translational regulation, CRMP2 activity is tightly controlled by phosphorylation and proteolysis. Either being phosphorylated by glycogen synthase kinase-3 beta (GSK-3β) at threonine 514 (T514) or processed by calpain to generate s-CRMP2 causes CRMP2 to lose its tubulin-binding activity [17,18]. GSK-3β-inactivated pCRMP2 is rapidly degraded, leading to suppressed axonogenesis in neuronal cells [19,20,21]. Intriguingly, no signal of CRMP2 T514 phosphorylation (pCRMP2) was detected (Figure 1b). Effects of lambda protein phosphatase (λPP) on CRMP2 profile were subsequently analyzed to probe if phosphorylation on other residues resulted in the CRMP2 profile. The CRMP2 pattern remained unaffected in the presence of λPP treatment (Figure 2b), indicating phosphorylation plays minor roles in the adipogenesis-coupled CRMP2 profile.

We further analyzed if calpain inhibitor ALLN treatment altered the CRMP2 pattern to examine possible proteolytic events. No prominent changes regarding CRMP2 profile were observed (Figure 2c), suggesting protein cleavage did not contribute to the identified CRMP2 pattern, either. Taken together, neither transcriptional control nor post-transcriptional protein modification/processing take part in the adipogenesis-coupled CRMP2 profile. Undetected pCRMP2 in the entire process revealed that CRMP2 activity is required for adipocyte differentiation; in addition, the rapidly down-regulated s-CRMP2 after the cells exited from the MCE phase further echoed our previous inference that s-CRMP2 modulates cell proliferation in the initial 48 hr after induction [7].

### 3.3. CRMP2 Colocalizes with Tubulin in Mitotic Clonal Expansion

**Figure 3:**
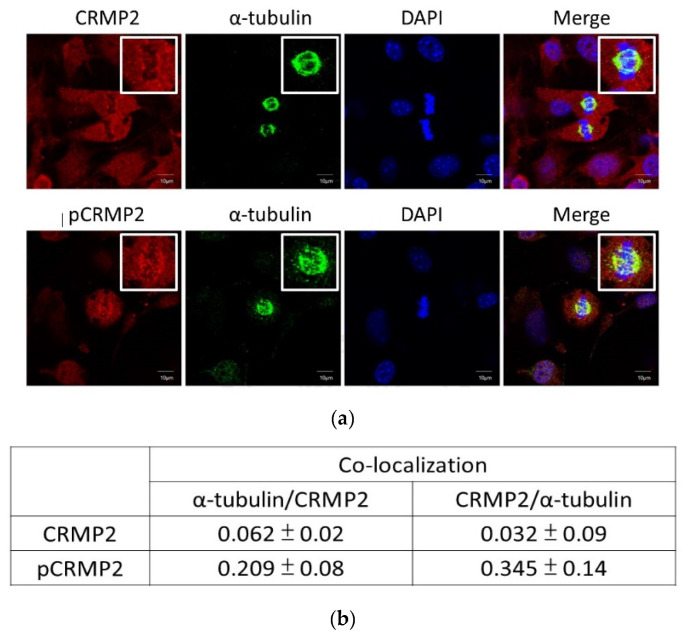
CRMP2 expression profile in mitotic clonal expansion phase. (**a**) Cells were induced into differentiation by MDI cocktail and subjected to immunostaining at 22 hr post induction. Images were obtained with Zeiss LSM700 confocal microscope using 63X objective lens, scale bar = 10 μm. (**b**) Quantification of confocal microscopic results in (**a**). Data are presented as means ± SEM of about 50 cells from different fields.

CRMP2 and pCRMP2 in cells in the MCE phase after 22 hr of induction were visualized by immunostaining to verify the participation of CRMP2 in cell proliferation. CRMP2 was ubiquitously dispersed in cytoplasm of nondividing cells, while colocalization of CRMP2 and α-tubulin at the mitotic apparatus was observed in cells undergoing mitosis (Figure 3). Significantly, a small amount of pCRMP2 was probed (thus probably not detectable by Western blotting), compared to CRMP2, and mainly co-localized with α-tubulin at the mitotic spindle. This observation supports the study by Lin et al. [22] that demonstrated binding of CRMP2 to tubulin during mitosis. In addition, it echoes our previous conclusion that CRMP2 is involved in adipogenesis by mediating cell proliferation at the MCE stage [7]. Therefore, MCE-coupled s-CRMP2 expression is required for cell proliferation, while it is rapidly degraded when the cells complete the proliferation task and exit from the stage. The mitotic spindle-associated pCRMP2 is speculated to mediate nuclear membrane disruption for the cells to construct mitotic apparatus in the peri-nuclear region. However, this speculation awaits further study.

### 3.4. CRMP2 Profile, GLUT4 Translocation and Lipid Droplets

Adipocytes are lipid reservoirs that store energy as triglycerides (TGs) in LDs [23]. CRMP2-knockdown cells hold smaller LDs and reduced insulin-promoted glucose uptake but significantly increased the amount of lipid deposits [7], indicating CRMP2 regulates glucose uptake and LD fusion. T514 de-phosphorylated CRMP2 exhibits tubulin-binding activity when either GSK-3β is inactivated by insulin [10] or cells are under confluence or quiescent state [24]. Therefore, putative effects of insulin on CRMP2 profile were investigated.

**Figure 4:**
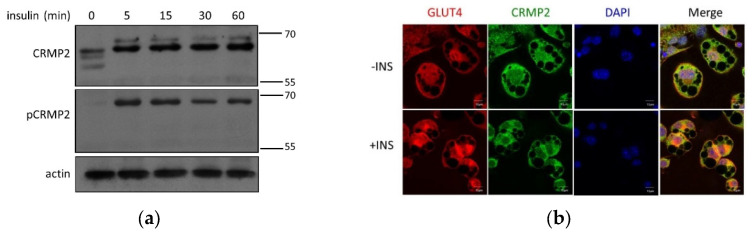

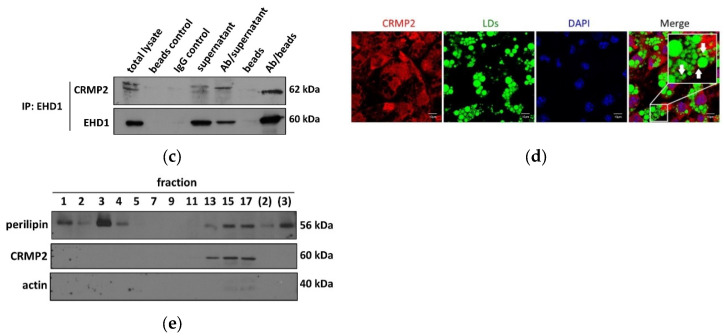
CRMP2 expression profile, GLUT4 and lipid droplets. (**a**) CRMP2 and pCRMP2 were analyzed by Western blotting in pre-adipocytes. (**b**) Confocal microscopy analysis of CRMP2 and GLUT4 localization in mature adipocytes without (-INS, upper panel) and with (+INS, lower panel) insulin treatment. (**c**) Anti-EHD1-precipitated protein complexes in adipocyte lysates were immunoblotted with anti-CRMP2 and anti-EHD1. (**d**) CRMP2 and LDs in adipocytes were analyzed by confocal microscopy. (**e**) Adipocyte lysates were subcellularly fractionated, followed by Western blotting using perilipin as LD marker. Fractions (2) and (3): white aggregates from fractions 2 and 3. Images were obtained with Zeiss LSM700 confocal microscope using 63X objective, scale bar = 10 μm (*n* = 3).

Regulation of pCRMP2 under insulin treatment was first analyzed in undifferentiated pre-adipocytes. While multiple CRMP2 subtypes were identified in nonconfluent cells without insulin signaling (Figure 1b), s-CRMP2 was rapidly diminished after 5 min of insulin treatment. Intriguingly, the insulin-promoted f-CRMP2 phosphorylation (f-pCRMP2) reached a climax at 15 min and slightly decreased thereafter (Figure 4a).

In mature adipocytes, CRMP2 and GLUT4 were ubiquitously distributed in cytosol in the absence of insulin treatment (Figure 4b, upper panel). CRMP2 was significantly decreased, and GLUT4 molecules were either translocated to plasma membrane or localized in peri-nuclear compartments under insulin treatment (Figure 4b, lower panel). Interestingly, co-localization between CRMP2 and GLUT4 implies the participation of CRMP2 in insulin-induced GLUT4 translocation. Therefore, it was tempting for us to examine if CRMP2 was associated with GLUT4 and the GSV trafficking/endocytosis mediator EHD1 [25,26,27]. Direct interaction between CRMP2 and EHD1 in adipocytes was identified (Figure 4c) despite no association detected between CRMP2 and GLUT4 (data not shown). The data suggest that CRMP2 is very likely to be involved in GLUT4 translocation via interaction with EDH1. Moreover, insulin-modulated CRMP2 expression and activity profile are very likely to be one of the contributing factors regulating glucose uptake ability via mediation of GLUT4 translocation.

Our previous study also suggested participation of CRMP2 in LD fusion; therefore, putative interactions between CRMP2 and LDs were visualized by immunostaining. Notably, CRMP2 puncta anchoring on LD surfaces were detected, supporting the involvement of CRMP2 in LD transport and/or fusion (Figure 4d). Nevertheless, no direct association between LDs and CRMP2 was identified in LD fractions (no. 1–4, Figure 4e) harvested by gradient centrifugation strategy, indicating either that CRMP2 does not directly bind to LDs or the binding is too weak to be remained and, therefore, detected during the experimental process.

### 3.5. Glucose Regulates CRMP2 Profile

The significant association between diabetes and lower CRMP2 in CRC patients suggests hyperglycemia modulates CRMP2 expression [8]. In this context, regulation of CRMP2 by glucose in adipogenesis was further investigated to address the effects of glucose on the CRMP2 profile. Pre-adipocytes were allowed to enter differentiation program in media containing either hyperglycemic (450 mg/dL or 25 mM, HG) or euglycemic (100 mg/dL or 5.5 mM, LG) glucose concentration. The CRMP2 expression profile was temporally analyzed in the whole period, and adipogenic efficiency was monitored by ORO staining.

**Figure 5:**
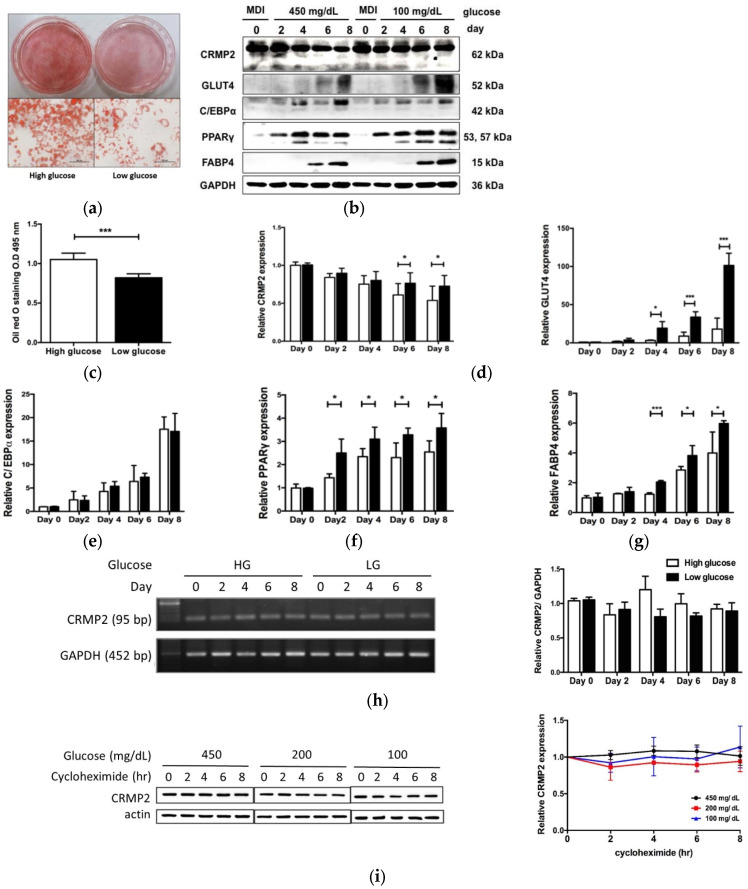
Effects of glucose on CRMP2 and adipogenic machinery. Cells were induced into differentiation in culture media containing either high (white bars) or low (black bars) glucose (*n* = 5). (**a**) Differentiation efficiency were determined by ORO staining, scale bar = 50 μm. (**b**) CRMP2 and important adipogenic proteins were analyzed by Western blotting. (**c**–**g**) Quantitative results of (**b**). (**h**) CRMP2 mRNA was analyzed by RT-PCR. (**i**) On day 4, cells were serum starved for 16 h, followed by cycloheximide (10 μg/mL) treatment and Western blotting (*n* = 3). * *p* < 0.05, *** *p* < 0.005 vs. HG.

Lipid contents in mature adipocytes differentiated in LG medium were significantly reduced by ~20% (Figure 5a) compared to the HG counterparts. CRMP2 was gradually decreased along with adipogenic progression in HG-treated cells (Figure 5b,c). Important adipogenesis-mediating genes C/EBPα and PPARγ, as well as adipocyte markers GLUT4 and FABP4, were all significantly elevated during the entire process, regardless of the glucose levels (Figure 5b–g). However, PPARγ, GLUT4, and FABP4 were significantly higher in euglycemic environments, compared to their levels in HG cells. CRMP2 under LG condition was also significantly higher than in HG cells in late phase (day 6 and 8). As CRMP2 mRNA (Figure 5h) and protein half-life (Figure 5i) remained unaltered in cells exposed to differential glucose concentrations, glucose does not affect transcriptional activity and post-translational protein degradation of CRMP2.

**Figure 6:**
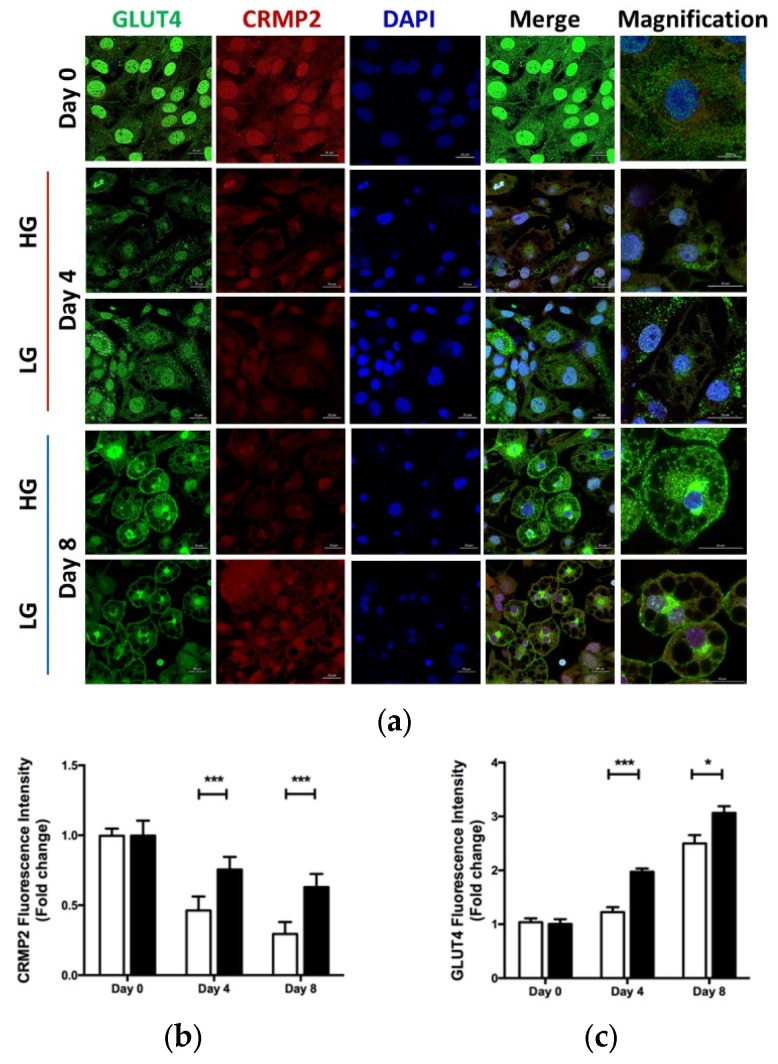
Effect of glucose on CRMP2 and GLUT4 profile. Cells were induced into differentiation in culture media containing either high (450 mg/dL, HG) or low glucose (100 mg/dL, LG). (**a**) CRMP2 and GLUT4 were detected by immunofluorescence at the indicated time. Quantitative results of CRMP2 (**b**) and GLUT4 (**c**) fluorescence intensity from (**a**). Images were captured using a Zeiss LSM 880 laser confocal microscope, and scale bars represent 20 μm or 10 μm (magnification). Data are presented as means ± SEM of about 50 cells in each group from 3 independent experiments. * *p* < 0.05, *** *p* < 0.005 vs. HG.

Intracellular levels and distribution of CRMP2 and GLUT4 were further investigated by immunostaining to visualize the down-regulatory effects of HG and GLUT4 on CRMP2 (Figure 6). Consistent with Figure 5b, hyperglycemic glucose significantly inhibited CRMP2 expression. Nevertheless, lipid contents of mature adipocytes under HG were significantly increased, probably due to ambient nutrient supply.

## 4. Discussion

### 4.1. CRMP2 Functional Profile in Adipogenesis

**Figure 7:**
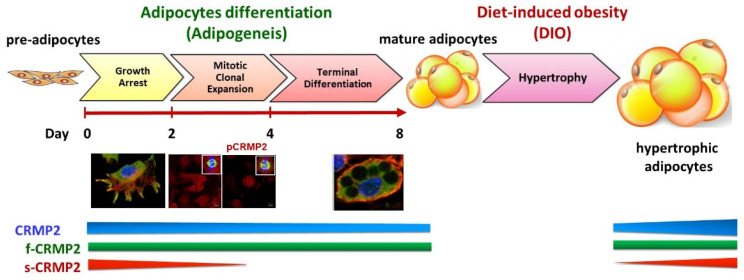
Implication of CRMP2 expression profile in physiological and pathological metabolic circumstances (scale bar = 10 μm).

Dramatic morphological changes and remodeling of gene expression are essential tasks for the fibroblastic pre-adipocytes to successfully transform into mature adipocytes. This adipogenic process is sequentially programmed and divided into three major phases, namely growth arrest (confluent) phase, mitotic clonal expansion (MCE) phase (cell proliferation), and terminal differentiation (adipocyte-destined) phase. As depicted in Figure 7, we concluded that the adipogenesis-coupled CRMP2 functional profile is implicated both in physiological energy homeostasis and pathological circumstances from DIO, insulin resistance and diabetes mellitus.

#### 4.1.1. Growth Arrest Phase

Reaching confluence to elicit contact inhibition in the initial growth arrest phase before entering differentiation is crucial for pre-adipocytes to efficiently undergo the differentiation scheme. At this stage, the pre-adipocytes have fibroblastic spindle morphology with multiple protrusions. CRMP2 is distributed mainly in the peri-nuclear region and protrusions (Figure 1a). The co-localization of CRMP2 and tubulin at the ends of protrusions implies CRMP2-tubulin interaction is necessary to maintain cytoskeleton stability for sustaining the fibroblastic shape. CRMP2 displays two major subtypes in this phase (Figure 1b), designated as f-CRMP2 (62~66 kDa) and s-CMRP2 (58 kDa), as described previously [7,18]. No pCRMP2 signal was detected, indicating that pCRMP2 is de-phosphorylated in response to contact inhibition-induced quiescence [7,28]. The s-CRMP2 was rapidly down-regulated and vanished within 5 min of insulin treatment, while phosphorylation of f-CRMP2 was promoted (Figure 4a).

#### 4.1.2. Mitotic Clonal Expansion Phase

Adding MDI cocktail allows the growth arrest cells to enter the MCE phase. The major missions in the MCE phase are to complete 2~3 rounds of cell proliferation and activate adipogenesis-driving machinery such as adipogenic transcription factor PPARγ within 48~72 h (Figure 1b). The s-CRMP2 was rapidly decreasing at this stage, leaving only f-CRMP2 detected after day 4. CRMP2 is evenly distributed in cytoplasm of non-dividing cells, whereas pCRMP2 is primarily enriched and co-localized with tubulin at the mitotic spindle in dividing cells (Figure 3). As pCRMP2 loses its tubulin-binding capacity to facilitate microtubule assembly [29,30], cytoskeletons with increased pCRMP2 in the perinuclear region have higher flexibility due to microtubule de-stabilization. Therefore, the findings imply inactive pCRMP2 assumes roles in the disruption of nuclear membrane for cells undergoing mitosis. Taking the above data and our previous study together [7], we concluded that the MCE-coupled s-CRMP2 overrides f-CRMP2 to direct cell proliferation and will be rapidly down-regulated in post-mitotic cells, while the mitotic spindle-associated pCRMP2 may mediate nuclear membrane disruption for the cells to construct the mitotic apparatus in the perinuclear region.

The above observations parallel the finding that s-CRMP2 takes the dominant role over f-CRMP2 to inhibit neurite elongation in neuronal development. The nuclear localization signal sequence is unmasked when the C-terminal domain of CRMP2 is processed to generate s-CRMP2 [18]; we speculate that s-CRMP2 may translocate into nucleus to trigger expression of gene sets required for cell division. Therefore, the CRMP2 profile is coupled with the status of cell confluence, proliferation and differentiation. On the other hand, the timeline for F-actin fiber de-polymerization within the initial 24 h after induction, followed by reorganization to form cortical F-actin structures within 48 h, coincides with MCE-coupled s-CRMP2 expression. Therefore, s-CRMP2 is also likely to mediate the dynamic actin remodeling events for the cells to compose mitotic apparatus at this stage.

#### 4.1.3. Terminal Differentiation Phase

The task of cells in this phase is to acquire characteristic features of mature adipocytes including the signature round-shape morphology, lipid synthesis and accumulation, and storage of lipids in LDs as lipid reservoirs. Fusion among smaller LDs for generating larger LDs and ultimately a single LD encompassing ~90% of intracellular space is continuously happening in this phase. Most of the cytoplasmic space in mature adipocytes is occupied by LDs on day 8 (Figure 1a). The CRMP2 level is significantly reduced, leaving f-CRMP2 as the only detected isoform and co-localized with tubulin at the rim of the cortical area facing intracellularly (Figure 1). High levels of adipocyte marker aP2 indicate the cells have completed the differentiation scheme and transformed into mature adipocytes.

In the scenario of axonal outgrowth, co-localization of CRMP2 with F-actin in the growth cones regulates axonal elongation by promoting microtubule assembly [9,31,32,33]. CRMP2-mediated actin dynamics are very likely to play vital roles in the dramatic morphological remodeling of the fibroblastic pre-adipocytes transforming to the characteristic round shape of mature adipocytes. At this stage, vanished s-CRMP2 allows f-CRMP2 to exert its functions of modulating glucose uptake, lipid synthesis/accumulation and morphological alterations for the cells to achieve the differentiation mission. Accordingly, manipulating the f-CRMP2/s-CRMP2 functional balance is suggested to control the body lipid reservoir and thus metabolic status via modulation of adipogenesis.

### 4.2. CRMP2 Function Profile in GLUT4 Transport and LD Fusion

Cytoskeleton plays an important role in the sequential events of GSV trafficking and GLUT4 translocation. Insulin signaling induces GSVs to move from the perinuclear area to the cell cortex along the microtubule bundles [34], followed by tethering, docking and the final fusion of GSVs with plasma membrane [35]. Cortical actin polymerization and remodeling are the rate-limiting steps for GSV fusion to plasma membrane [36]. Impaired actin remodeling interferes with GSV fusion events despite insulin-induced GSV transport to the cortical area remaining intact. Insulin-enhanced CRMP2-GLUT4 co-localization in mature adipocytes suggests the involvement of CRMP2 in GSV trafficking (Figure 4b). Combining the evidence from our previous study, we suggest that CRMP2-GLUT4 co-localization is involved in GSV transport and fusion via mediation of cortical F-actin polymerization, which subsequently affects glucose uptake efficiency in an insulin-independent manner [7].

The differentiation-dependent CRMP2 expression profile is also well-characterized in neuronal polarization and axonogenesis of hippocampal neurons [37]. CRMP2 performs critical functions in endocytosis and protein trafficking during axon elongation and formation, including the endocytosis-mediated recycling of neuronal cell adhesion molecule L1 through interaction with Numb [9,38], and the transport of kinesin-1 to the distal part of growing axon via linking of kinesin-1 to microtubule [39,40].

We previously demonstrated that CRMP2 siRNA-transfectants show disturbed GLUT4 translocation and LD fusion due to suppressed actin polymerization [7], indicating that cortical F-actin meshwork is required for GLUT4 translocation. Similarly to GSV trafficking, small LDs move along microtubules and fuse with each other to form larger LDs during the terminal differentiation phase [41]. Our study reveals that both glucose uptake and LDs fusion are significantly affected once the balance of CRMP2-mediated cytoskeleton dynamics is impaired. As polarized endocytosis and membrane recycling play crucial roles in generating neuronal polarity, and GSK-3β-induced pCRMP2 loses its binding activity to tubulin and Numb [10,40], we speculate that CRMP2 functions in GSV trafficking/recycling and LD fusion through cytoskeleton remodeling and Numb-mediated endocytosis during adipogenesis.

Taken together, our studies suggest insulin enhances GSV translocation from the perinuclear region to the juxta-plasma membrane area, followed by CRMP2-mediated insulin-independent cytoskeleton re-organization to facilitate GSV–plasma membrane fusion, which allows the insertion of GLUT4 molecules into cell surface to perform their function of glucose uptake. In addition, the identified CRMP2 puncta on the LD surface and the association between EHD1 and CRMP2 further imply the participation of CRMP2 in LD fusion. Nevertheless, no putative association between LD surface protein perilipin and CRMP2 was found, probably due to weak binding force or indirect interaction between CRMP2 and LDs.

### 4.3. Glucose Regulates CRMP2 Functional Profile

Compared with CRC patients, tumor lesions in subjects with CRC-DM comorbidity have significantly lower CRMP2, which is associated with advanced tumor stage [8]. Phosphorylated f-CRMP2 (f-pCRMP2) is the major CRMP2 isoform identified in CRC tumor lesions while s-CRMP2 (s-pCRMP2) is the main isoform in CRC-DM subjects. MCE phase-coupled s-CRMP2 expression (Figure 1) further echoes the conclusion that s-CRMP2 links to cell proliferation as evidenced in several cancers [8]. The association between diabetes and CRMP2 in CRC patients suggests hyperglycemia mediates the CRMP2 profile. Here, we demonstrate that hyperglycemia-inhibited CRMP2 results in higher cytoskeletal flexibility and contributes to CRC disease progression. In brief, we add evidence illustrating diabetic hyperglycemia-suppressed CRMP2 in CRC tumorigenesis and disease progression. In this context, glucose determines cancer cell proliferation and malignant traits via manipulation of CRMP2 function profiles and thus the cytoskeleton dynamics. Accordingly, it is worth probing the effects of glucose on CRMP2 in adipogenesis for further picturing the physiological and pathological roles of CRMP2 in lipid metabolism and energy homeostasis.

In support of the previous findings that CRMP2 is significantly decreased in CRC patients with diabetic comorbidity, we documented that hyperglycemia inhibits CRMP2 expression and suppresses adipogenic machinery and adipocyte markers (Figure 6). Intriguingly, cells manifest significantly lower GLUT4 levels and smaller LDs may probably result from the inefficient LD fusion caused by suppressed CRMP2 function. However, cell under HG treatments store higher amounts of lipids, implying hyperglycemia provides massive nutrient supply to compensate for the consequences that result from the down-regulated CRMP2 profile.

### 4.4. Significance of Maintaining CRMP2 Function Profile in Adipogenesis

CRMP2 activity is tightly controlled by phosphorylation. Dominant negative CRMP2 mutants suppress axon formation by overriding endogenous CRMP2 activity. Approximately one-third of CRMP2 molecules are phosphorylated at T514, which are enriched in growing axons but not at the growth cones of cultured hippocampal neurons [10]. This indicates a substantial portion of CRMP2 binds to tubulin in hippocampal neurons. Active GSK-3β impairs neuronal polarization, while non-phosphorylated CRMP2 counteracts the inhibitory effects of GSK-3β. Likewise, opposite signals from s-CRMP2/f-CRMP2 and phosphorylation/de-phosphorylation of the tubulin-binding determinant T514 must maintain a sophisticated balance both spatially and temporarily throughout the entire adipogenic process for the cells to complete the mission of acquiring adipocyte features. In this context, an imbalance of the CRMP2 functional profile leads to inefficient differentiation originated from disturbed cell proliferation in the MCE phase, down-regulated critical adipogenic transcription factors and lipogenic enzymes, reduced glucose uptake based on impaired GSV fusion and lack of unilocular LD due to impaired LD fusion. The net consequences of the above phenotypes are impaired glucose uptake, insulin resistance, and eventually metabolic disorders.

Balance of f-CRMP2/s-CRMP2 and the corresponding phosphorylated status must be finely controlled to properly adjust the cytoskeletal stability for meeting the dynamic cellular needs. Manipulating the s-CRMP2/f-CRMP2 ratio and thus the cytoskeleton dynamics is anticipated to improve the efficiency of glucose uptake and insulin sensitivity. Moreover, facilitating cortical F-actin arrangement in adipocytes is suggested to enhance cellular mechanical strength and, therefore, lessen adipocyte hypertrophy in diet-induced obesity [41]. In brief, our data provide molecular evidence explaining the functions of CRMP2 in physiological, pathological and disease progression in metabolic homeostasis and disorders related to metabolic abnormalities, including cancer.

### 4.5. CRMP2 Functional Profiles in Pathological Events

We previously disclosed the association between CRMP2 with obesity and metabolic disorders by evidencing significantly increased CRMP2 in adipose tissues of diet-induced obese (DIO) mice, probably due to chronic inflammation induced by insulin resistance [7]. Notably, diabetic onset prominently attenuates the enhanced CRMP2. The highly expressed s-CRMP2 in DIO may play certain roles during the process of developing obesity until diabetic onset, and then be attenuated by hyperglycemia. Moreover, CRC with reduced CRMP2 levels and activity under a long-term hyperglycemic environment tends to have higher metastatic potential and advanced staging due to cytoskeleton instability, lower stiffness and loose cell–cell adhesion [8]. A growing body of evidence documents the participation of s-CRMP2 in tumorigenesis and thus its association with poor prognosis in various cancers [7,28,42,43]. Collectively, these findings address the perspective of considering CRMP2 as a CRC biomarker [44] and its correlation with poor cancer prognosis [10,11,45]. The current study provides further evidence that metabolic alteration-disturbed homeostasis of CRMP2-associated cytoskeleton dynamics participates in pathogenesis, leading to metabolic and cancerous consequences.

### 4.6. Unanswered Questions

It is tempting for us to uncover the underlying factors explaining the adipogenesis-coupled CRMP2 profile and the regulatory mechanism of glucose to CMRP2, as well as the mechanism responsible for s-CRMP2 alterations from physiological adipogenesis, DIO and diabetes mellitus. Nevertheless, neither transcriptional nor post-translational modification, at least phosphorylation and proteolysis, contributes to the differential CRMP2 expression during adipocyte differentiation or regulatory effects of glucose on CRMP2 in mature adipocytes. These mysteries await further study.

Another CRMP2 subtype with molecular mass higher than that of f-CRMP2 was probed in ALLN-treated cells (Figure 2) and insulin-treated pre-adipocytes (Figure 4a). A 75-kDa CRMP2 isoform corresponding to the long N-terminal splice variant is described in embryonic, neonatal and adult rat brain [18,46]. It is intriguing to examine the identity and function of this uncharacterized CRMP2 subtype for drawing a clearer contour of CRMP2 in adipocyte differentiation and energy metabolism.

Pathologically, enhanced nuclei-localized pCRMP2 is implicated in disease progression and associated with lower survival rates of cancer patients [13,47,48]. We hence suggest that pCRMP2 levels and nuclear localization, instead of the total CRMP2 amounts, should be the study focus in delineating CRMP2 functions. However, the exact CRMP2 isoform co-localized with tubulin at the mitotic spindle of dividing cells (Figure 3) needs further investigation since the antibodies used were not able to differentiate f-CRMP2 from s-CRMP2.5.

## 5. Conclusions

Adipogenesis and lipid metabolism profoundly affect metabolic homeostasis and energy reservoirs. Interfering with metabolic balance is closely associated with multiple disorders, thus, understanding the sequential events of adipocyte differentiation holds the keys to developing a efficient strategy of tackling the globally increasing trend of obesity and related diseases.

Originally well-characterized as the determinant of axonogenesis in neurons, emerging evidence indicates that CRMP2 is involved in multiple physiological activities, including apoptosis/proliferation, cell migration and differentiation. Here, we provide novel clues illustrating the contributions of CRMP2 to dramatic morphological change from pre-adipocyte to mature adipocyte, as well as the fusion of GSVs to plasma membrane and between LDs during the transformation of pre-adipocytes to mature adipocytes via control of cytoskeleton dynamics.

In addition, insulin and glucose are characterized as independent signals that regulate adipogenesis via mediation of the CRMP2 profile. Our study reveals that the CRMP2 functional profile is not only coupled with the adipogenesis scheme, but also implicated in obesity, metabolic imbalance and CRC disease progression in diabetic patients. Therefore, CRMP2 plays important roles in the physiopathology regarding the etiology and progression of multiple diseases, rather than being limited to neurodegenerative disorders. However, more questions are thus raised and must be answered to illustrate the whole picture concerning CRMP2 functions in metabolic homeostasis, such as post-translational modifications other than phosphorylation during adipogenesis. A better understanding of CRMP2 roles and mechanisms of action in physiological and pathological evolution of related diseases is crucial.

## Data Availability

Not applicable.
